# Let us talk about death: gender effects in cancer patients’ preferences for end-of-life discussions

**DOI:** 10.1007/s00520-019-05275-1

**Published:** 2020-01-18

**Authors:** C. Seifart, J. Riera Knorrenschild, M. Hofmann, Y. Nestoriuc, W. Rief, P. von Blanckenburg

**Affiliations:** 1 Faculty of Medicine, Research Group Medical Ethics, AGEM, Philipps-University of Marburg, Baldingerstraße 1, 35043 Marburg, Germany; 2 Department of Internal Medicine, Division of Haematology and Oncology, Philipps-University of Marburg, Marburg, Germany; 3 Department of Clinical Psychology and Psychotherapy, Philipps-University of Marburg, Marburg, Germany; 4 Clinical Psychology, Helmut-Schmidt-University Hamburg, Hamburg, Germany

**Keywords:** End-of-life discussions, Gender effects, Palliative care, Communication, Cancer

## Abstract

**Purpose:**

Patients with advanced cancer often receive suboptimal end-of-life (EOL) care. Particularly males with advanced cancer are more likely to receive EOL care that is more aggressive, even if death is imminent. Critical factors determining EOL care are EOL conversations or advance care planning. However, information about gender-related factors influencing EOL conversations is lacking. Therefore, the current study investigates gender differences concerning the content, the desired time point, and the mode of initiation of EOL conversations in cancer patients.

**Methods:**

In a cross-sectional study, 186 female and male cancer patients were asked about their preferences for EOL discussions using a semi-structured interview, focusing on (a) the importance of six different topics (medical and nursing care, organizational, emotional, social, and spiritual/religious aspects), (b) the desired time point, and (c) the mode of discussion initiation.

**Results:**

The importance of EOL topics differs significantly regarding issue (*p* = 0.002, *η*^2^ = 0.02) and gender (*p* < 0.001, *η*^2^ = 0.11). Males wish to avoid the engagement in discussions about death and dying particularly if they are anxious about their end-of-life period. They wish to be addressed regarding the “hard facts” nursing and medical care only. In contrast, females prefer to speak more about “soft facts” and to be addressed about each EOL topic. Independent of gender, the majority of patients prefer to talk rather late: when the disease is getting worse (58%), at the end of their therapy, or when loosing self-sufficiency (27.5%).

**Conclusion:**

The tendency of patients to talk late about EOL issues increases the risk of delayed or missed EOL conversations, which may be due to a knowledge gap regarding the possibility of disease-associated incapability. Furthermore, there are significant gender differences influencing the access to EOL conversations. Therefore, for daily clinical routine, we suggest an early two-step, gender-sensitive approach to end-of-life conversations.

## Introduction

A high quality of end-of-life care and a “good death” as part of an improved patient-centered care at the end-of-life (EOL) have become important goals of medical treatment [1, 2]. These goals include receiving adequate pain and symptom management, retaining control, and choosing the place where dying and death occur and/or who will be present, thereby strengthening relationships with loved ones, having time to say goodbye, and choosing not to have life-prolonging medical interventions [2, 3]. Quality of life and physical distress directly correlates with the received medical care in the last period of life. More aggressive end-of-life care, such as ICU admission or starting new chemotherapy regimens and less hospice care, is associated with impaired quality of life [4–8]. Especially patients with advance cancer often receive suboptimal end-of-life care [9, 10].

The main factor for receiving EOL care consistent with patients’ wishes or preferences is end-of-life discussion (EOLD) with the attending physician [11, 12], which is also associated with less aggressive end-of-life care. However, the required end-of-life conversations including their formalization are challenging and difficult to conduct. Although it is recommended to start EOLD early, physicians are often uncertain as to the optimal time point or content of EOL discussions [13]. Approximately only 30–40% of terminally ill patients had discussed their end-of-life decisions with their physicians [6, 13, 14]. EOL discussions have therefore often been reported to take place when a medical crisis arises, at hospitalization for severe progression even at the day of death [14–16]. Besides, in the last decades, it has become evident that it does not suffice to solely focus on the questions of “do not resuscitate” and on the “product” of advanced directives (AD) [11]. Instead, EOL discussions have experienced a shift towards a conversation process [13, 14, 17], better known as *advance care planning* (ACP). These conversations help patients to formulate goals of care, clarify value sets and wishes to design a corresponding care plan [6, 7, 15]. However, the approach to end-of-life discussions is still challenging. To make matters even more complex, recent research points to gender effects in end-of-life care. Particularly males with advanced cancer are more likely to receive ICU admission, more chemotherapy, and less hospice care near death [18]. Males reporting EOL discussions were less likely to experience an ICU stay compared with those without EOL discussions, while this effect was not observed for females [18]. In the Canadian Study of Health and Aging, female gender was associated with giving thoughts to and having discussion about end-of-life preferences [19].

Although EOL discussions are correlated with consistent and less aggressive EOL care, information about the influence of gender differences on these conversations is very limited. In particular, scientifically sound information about gender differences in preferences for content, timing, and initiation of end-of-life discussions is lacking.

Therefore, the present study investigates gender differences in cancer patients regarding preferences concerning content, optimal time point, mode of initiation, and dialog partners for EOL discussions. To that end, a new, semi-structured interview was developed. The interview focused on the importance patients give to different end-of-life topics including medical care, nursing care, organizational and social aspects, spirituality and emotions, the desired time point of the conversation, and the mode of initiation. Since information regarding the optimal timing of EOL conversations is still a matter of debate, we included cancer patients in an “early” and “late” stage of disease (rehabilitative and advance).

## Methods

### Participants and design

In addition to written informed consent, inclusion criteria for all patients were the diagnosis of malignancy, sufficient German language skills, and a minimum age of 18. Patients were recruited in a palliative in- and out-patient setting of the University Hospital Marburg (UKGM), Germany, and in a rehabilitation clinic (Klinik Sonnenblick) in Marburg, Germany. After being informed about the study and giving a written informed consent, patients were interviewed by five psychological master students and medical students under supervision of CS, YN, MH, and PvB. Interviews lasted 45–90 min.

### Ethics

The study was approved by the institutional review board of the Medical School, Philipps-University of Marburg. Before participation, subjects gave written informed consent.

### Assessment instruments

Besides assessing socio demographical and clinical variables, patients were interviewed with a semi-structured interview. They were asked questions about six topics that play a role in EOL situations. The selection of the topics was based on literature and discussion with experienced clinicians.

1. Medical care: e.g., treatment of physical symptoms (e.g., dyspnea, sickness, or pain), advanced directives, life-extending measures
2. Nursing care: e.g., nursing care in the last period of life and in the actual dying phase, place of dying
3. Organizational aspects: e.g., financial and legal issues like pension, inheritance, or funeral
4. Emotions: e.g., grief, anger, fear, worries
5. Social aspects: e.g., dealing with relatives, saying goodbye, unresolved conflicts
6. Religiosity/spirituality: e.g., religious beliefs or desires, thoughts about death and the hereafter

For each of these areas, patients were asked, among other questions, *how they rated their individual importance to talk* about the given topic (Likert scale from 1 (“not at all”) to 10 (“very much”)), if they *want to be addressed* to talk about the topic, and *when* they want to talk about each topic ((1) disclosure/beginning of therapy, (2) end of therapy/self-sufficiency, or (3) disease getting worse). Moreover, patients were asked if they have an AD (“yes”/“no”) and if they have already talked about end-of-life themes (Likert scale from 1 (“very often”), 2 (“often”), 3 (“from time to time”), 4 (“scarce”) to 5 (“never”)). They were asked if they want to avoid any engagement in end-of-life issues (Likert scale from 1 (“not at all”) to 5 (“very much”)) and if they have fears of their ends of life (Likert scale from 1 (“not at all”) to 5 (“very much”)).

Mental and physical quality of life was assessed using the 12-item Short-Form Health Survey (SF-12) [20] and patients’ performance status (Karnofsky performance status scale) by the attending physician.

### Data analysis

Analyses were performed using SPSS 24 (SPSS Inc. IBM, Chicago, IL, USA), with statistical significance set at *p* < 0.05. For the analyses, the sample of *n =* 175 patients will allow the detection of small to medium effect sizes with 80% power and *α =* 0*.*05. Data were screened for univariate outliers, missing data, and violations to the assumptions of analyses. Missing data at random (2.23%) were imputed using multiple imputations. To compare both genders regarding their preferences for end-of-life discussions, and to control possible influences of demographic and clinical characteristics, (multivariate) analyses of covariance (MANCOVA and ANCOVA) were conducted. For categorical data, chi-square tests were used for comparison of gender concerning demographic and medical variables. Pearson correlations were used to analyze relations between variables. Further details will be reported in the “Results” section.

## Results

### Patients

**Fig. 1:**
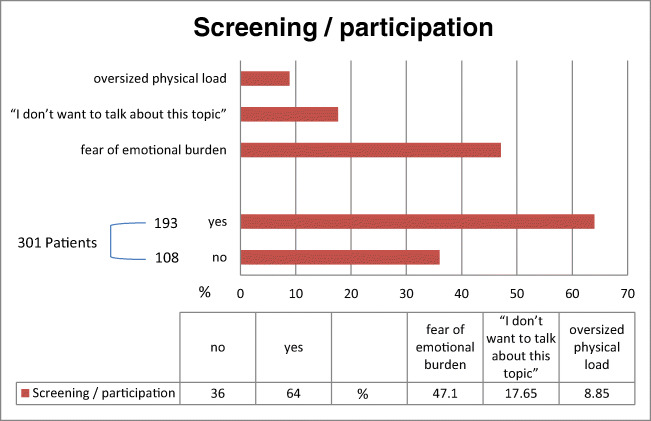
Refusal rates and reasons

Of 301 patients who were asked to participate, *N* = 193 (64%) agreed to do so. The most common reasons for refusal (*n* = 108, 36%) were the following: fear of emotional burden (47.1%), “I don’t want to talk about this topic” (17.65%), and oversized physical load (8.82%) (see Fig. 1). There were no significant gender differences in participation/refusal rate (55% female) or in reasons for refusal. Seven patients did not finish the interview and were therefore excluded from analysis. Thus, 186 patients were analyzed. Detailed demographic and clinical characteristics of the study population are listed in Table 1.

**Table 1:** Demographic and medical characteristics of cancer patients

	Males (*n* = 92)	Females (*n* = 94)	*p* values^a^
Age, *M* (*SD*)	61.9 (11.9)	58.7 (11.8)	*p* = 0.078
Marital status, *n* (%)			
Unmarried	9 (9.8)	9 (9.6)	
Married	66 (71.1)	53 (56.4)	
Divorced	11 (12.0)	14 (14.9)	
Widowed	6 (6.5)	18 (19.1)	*p* = 0.051
Children, *n* (%)			
Yes	78 (84.8)	83 (88.3)	
No	14 (15.2)	11 (11.7)	*p* = 0.48
Years of education, *n* (%)			
≥ 13	15 (16.5)	9 (9.7)	
≥ 10	16 (17.9)	32 (34.4)	
9–10	59 (64.8)	52 (55.9)	
No graduation	1 (1.1)	0 (0)	*p* = 0.041
Setting, *n* (%)			
Palliative	56 (60.9)	29 (30.9)	
Rehabilitation	36 (39.1)	65 (69.1)	*p* < 0.001
Type of cancer, *n* (%)			
Gynecological	1 (1.1)	46 (48.9)	
Bronchial	25 (27.2)	9 (9.6)	
Glioblastoma	21 (22.8)	12 (12.8)	
Hematologic	13 (14.1)	10 (10.6)	
Urogenital	9 (9.8)	4 (4.3)	
Other (each *n* < 7)	16 (17.4)	10 (10.6)	*p* < 0.001
Karnofsky, *M* (*SD*)	73.5 (13.5)	78.0 (13.9)	*p* = 0.046
SF-12, *M* (*SD*)			
Physical QOL	36.0 (10.1)	38.8 (10.1)	*p* = 0.057
Mental QOL	50.4 (10.3)	50.5 (10.5)	*p* = 0*.*945

^a^*t* test for age, Karnofsky, and SF-12; chi-squared-tests for sex, marital status, years of education, and type of cancer

*SF-12* Short-Form Health Survey, *Karnofsky* Karnofsky performance status scale, *QOL* quality of life

### Did they talk about their own end-of-life period or have an AD?

In chi-square tests, they were no gender differences in having an AD in our sample: 32.6% of the males and 29.8% of the females had an AD (*p* = 0.537). Moreover, there were no differences in having an AD between the persons of different settings.

In a MANOVA, there were differences between the disease setting and the frequency of discussions in having talked about death (*F*(1,180) = 15.105, *p <* 0*.*001, partial *η*^2^ = 0.08): 47.1% of the patients from the palliative setting had no or scarce conversations about their own EOL period, and 64.4% of the patients from the rehabilitation setting had no or scarce conversations. Gender differences were not found regarding the frequency of conversations about death (*p* = 0.518). However, females were less likely to avoid talking about issues of death and dying during their illness (*F*(1,180) = 4.959, *p* = 0.027, partial *η*^2^ = 0.03), even if they tended to have more EOL fears (*F*(1,180) = 3.597, *p* = 0.053, partial *η*^2^ = 0.02). Thus, 51.6% of the females reported having EOL fears “partly” to “very much” versus 36.3% of the males, whereas 33% of the females reported the wish to avoid the engagement in EOL issues “partly” to “very much” versus 50.0% of the males.

There was a significant correlation between end-of-life fears and the tendency to avoid EOL conversations (*r* = 0.164, *p* = 0.026) independent of gender. However, males reported less conversations about death and dying if they were anxious about their own end-of-life (*r* = − 0.215, *p* = 0.042).

There were no significant correlations between conducting of an EOL conversation and other medical or demographic variables (e.g., education, the self-rated quality of life) except of the Karnofsky index. Patients with a lower Karnofsky index were more likely to have had an EOL conversation (*r* = 0.201, *p* = 0.014). A higher age was related to less end-of-life fears (*r* = − 0.187, *p* = 0.015).

Nearly all participants agreed that self-determination is of distinct importance to them (palliative patients 95.5%, rehab patients 95%).

### What they want to talk about—importance to speak about specific topics

All patients were asked to rate the importance of the six EOL topics. A mixed design analysis of covariance (ANCOVA) with the six end-of-life topics as within-factor; gender as between-factor; and setting, age, and quality of life as covariates was conducted. The results showed significant differences in the importance of topics (*F*(5,855) = 3.48, *p* = 0.004, partial *η*^2^ = 0.02). Most importantly, patients wanted to talk about their medical care (*M* = 8.21, SD = 2.17) and organizational aspects (*M* = 8.01, *S* = 2.78). At the bottom of the list were religious or spiritual topics (*M* = 4.84, *SD* = 3.28). The patients were asked if they had already talked about the different topics. About half had talked about organizational aspects (50.5%), followed by medical care (44.6%), emotions (39.2%), nursing care (28.5%), social aspects (21.0%), and spirituality/religiosity (20.4%). Females stated to have had significantly more discussions about nursing care (*χ*^2^ *=* 7.54, *p* = 0.006) and social aspects (*χ*^2^ *=* 8.05, *p* = 0.003).

**Fig. 2:**
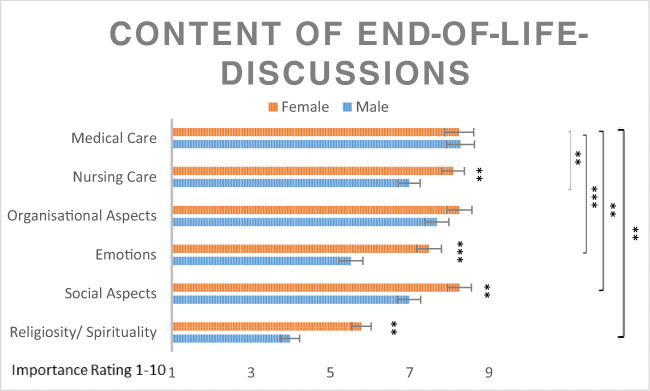
Importance ratings for the content of end-of-life discussions. Values show the means and standard errors (*n =* 179)*.* ***p < 0.001, **p < 0.01

There was a significant main effect of gender (*F*(1,173) = 20.21, *p* < 0.001, partial *η*^2^ = 0.11), showing that females rated the topics as more important. The significant interaction between gender and topic (*F*(5,865) = 5.86, *p* = 0.001, partial *η*^2^ = 0.02) indicated with contrasts that females want to speak more about nursing care (*F*(1,173) = 6.83, *p* = 0.01, partial *η*^2^ = 0.04), emotions (*F*(1,173) = 14.5, *p* < 0.001, partial *η*^2^ = 0.08), social aspects (*F*(1,173) = 8.56, *p* = 0.004, partial *η*^2^ = 0.05), and religiosity/spirituality (*F*(1,173) = 10.7, *p* = 0.001, partial *η*^2^ = 0.06) than males (see Fig. 2). Both genders had the same interest in speaking about medical care and organizational aspects. The covariates setting, quality of life, and age showed no differences in the topics. Means, standard errors, and results of contrasts are shown in Fig. 1. All results stayed stable after controlling for the possible confounders Karnofsky index, setting, education, and type of cancer*.*

Based on their content and statistical proximity and for the purpose of improved clarity, the indicated topics will be summarized for the following analyses: (1) medical and nursing care, (2) organizational aspects, (3) emotional and social aspects, and (4) religiosity/spirituality.

### When they want to talk—desired time to talk

**Fig. 3:**
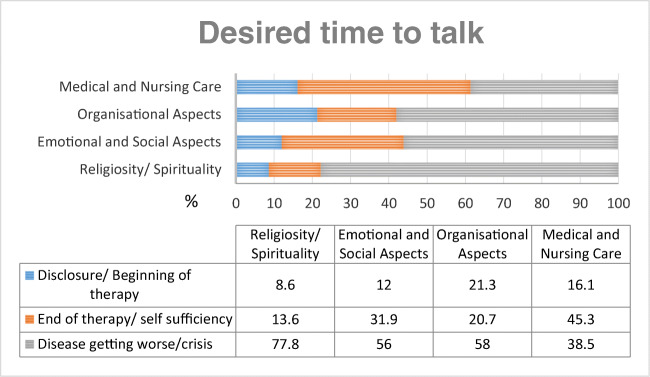
Percentage of the desired time to talk about specific topics (*n* = 161 (medical and nursing care)*–n* = 169 (organizational aspects))

Cancer patients rated when they want to talk about specific topics (“disclosure/beginning of therapy,” “end of therapy/self-sufficiency,” or “on demand/disease getting worse/crisis”). In total, the majority of the interviewed cancer patients would like to talk about any topic when their disease is getting worse (58%); 27.5% prefer to talk at the end of therapy or end of self-sufficiency, and only 14.5% of the patients want to talk at the disclosure or the beginning of therapy. Percentages of ratings when cancer patients want to talk about a specific topic are illustrated in Fig. 3. In chi-square tests, there were no differences between setting and gender in the desired time to talk.

### Should the EOL topics be addressed to the patients?

**Fig. 4:**
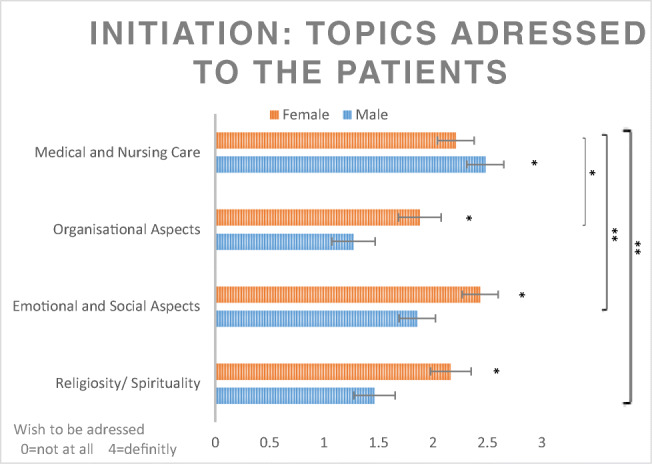
Patients want to be spoken or not referring to different topics. Values show the means and standard errors (*n* = 141). **p<0.01, *p<0.05

Patients rated on a 5-point Likert scale if they wanted to be spoken to (= 4) or not (= 0). A mixed design analysis of covariance (ANCOVA) was used with the four end-of-life categories as within-factor, gender as between-factor, and setting, age, and quality of life as covariates. The results showed a significant main effect of gender (*F*(1,135) = 5.63, *p* = 0.02, partial *η*^2^ = 0.04) and a significant interaction between topic and gender (*F*(3,405) = 3.85, *p* = 0.010, partial *η*^2^ = 0.03) indicating that males and females differ in their wish to be contacted. Contrasts revealed that males prefer to be addressed about nursing and medical care only, whereas females also want to be approached regarding all topics including organizational aspects (*F*(1,135) = 5.39, *p* = 0.002, partial *η*^2^ = 0.07), emotional and social aspects (*F*(1,135) = 9.02, *p* = 0.003, partial *η*^2^ = 0.06), and religiosity (*F*(1,135) = 9.80, *p* = 0.002, partial *η*^2^ = 0.07). No effects of setting, age, or quality of life were found. Means, standard errors, and results of contrasts are shown in Fig. 4. All results stayed stable after controlling for the possible confounders, Karnofsky index, setting, education, and type of cancer.

### To whom the patient wants to talk to?

**Fig. 5:**
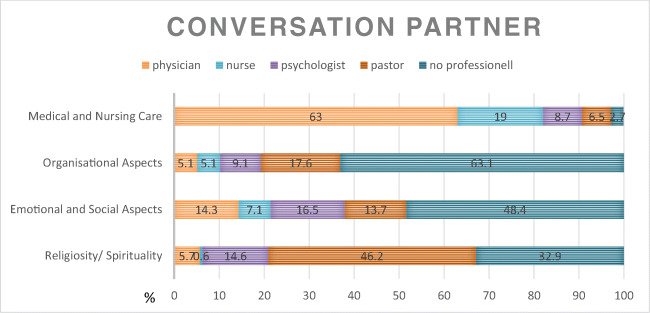
With whom of the professionals patients want to speak about the different topics (*n* = 158 (religiosity)–*n* = 184 (medical and nursing care))

Patients stated to whom they had already talked about end-of-life issues: 49.5% had talked to family members, 42.5% with their partners, 28% with friends, 18.3% with a physician, 7.5% with other cancer patients, 5.4% with a psychologist, and 2.2% with a priest/pastor or spiritual person. Females reported significant more EOL discussions with a psychologist (*χ*^2^ = 7.54, *p* = 0.006) and other cancer patients (*χ*^2^ = 8.05, *p* = 0.003) than males. With whom of the professionals patients want to speak about the different topics is shown in Fig. 5.

## Discussion

The study investigated gender differences in cancer patients’ preferences concerning the optimal time point, content, and mode of initiation for EOL discussions. Overall, the majority of the patients wished to talk rather late about EOL issues independent of gender or disease stage. Beyond that, there is a main effect of gender concerning approach, content, and handling of these conversations.

### Timing of end-of-life discussions

Similar to results published by others [6, 7, 16, 21–23], we found a low frequency of reported EOL conversations. Otherwise, patients stated a high interest in discussing end-of-life issues, especially medical, organizational, and nursing aspects and a very high desire for self-determination. This contrast might result from the intention to postpone these conversations to a later time point. The majority of our patients wished to talk about end-of-life issues at the end of therapy or at the occurrence of a (serious) crisis. This wish to shift EOL discussions to the latest possible time may be primarily due to sociological and individual psychological factors. Sociological barriers originate from the general intention to avoid topics such as death and dying [24] in society, whereas individual psychological factors comprise concerns and fears, the intention to protect family members from straining conversations and “magic” thinking (“if I talk about it, it comes true”). In the present study, there was a significant correlation between end-of-life fears and the tendency to avoid EOL conversations independent of gender, pointing to the importance of individual psychological barriers. However, another key factor contributing to the strong tendency to shift EOL discussions is that patients misconceive the expected time between the end of therapy and the terminal phase of disease and the risk of unexpected severe disease progression. It is known that physicians’ prediction of an individual patient’s prognosis is often imprecise [25–27], and prognosis is only discussed limited [16, 25]. Moreover, patients with advanced cancer have often limited understanding of the incurability of their disease and substantial misconceptions regarding prognosis that result in dramatically overestimation of survival time [28–31]. However, it is not exclusively a question of quantitative estimation of survival. The capacity to express wishes and values is also of distinct importance. One of the main barriers for discussing goals of care or end-of-life decisions is the lack of capacity of the person concerned [22, 23]. In advanced cancer, disease progression can be unexpected and serious crises are often associated with cognitive impairment. However, these aspects are often not considered in patients’ information about the course of the disease. To make matters worse, physicians hesitate to initiate EOL conversations due to fears of harming patients’ feelings or destroying hopes while also waiting for patients’ initiation [32, 33], aggravating the tendency to talk late.

Although the patients’ intention to talk rather late should be respected and a postponement might be psychologically beneficial, it has to be considered that a delay might contradict the patients’ wish for self-determination at the EOL thereby enhancing the risk to receive inconsistent EOL care. Albeit patients should not be enforced into EOL conversations, there is a clear need to inform about the consequences of unexpected disease deterioration and related incapacity to express wishes and values.

### Gender effects

Contrary to the patients’ preferences for timing, the approach to and the content of EOL conversations differ significantly by gender. Males stated more frequently that they wish to avoid the engagement in issues of death and dying, particularly if they were anxious about their own end-of-life. In contrast, females were less likely to avoid talking about issues of death and dying during their illness, even if they tended to have more EOL fears. Besides, females rated nursing care and “soft” topics, such as emotional, social, and religious aspects, as more important than males and talked more frequently about organizational aspects and nursing care. Males prefer to be addressed exclusively about “hard fact” topics, e.g., medical care and organizational aspects, while females want to talk about all topics, reflecting an easier approach to EOL conversations for females. Therefore, based on the known association between EOL discussions and less aggressive and more consistent EOL care, it can be argued that males are at higher risk of non-beneficial EOL care. In fact, males having not discussed end-of-life care were significantly more likely to receive ICU care in their last week of life [18]. Actually, there are no studies investigating gender effects concerning cancer patients’ preferences for the approach to and content of EOL conversations. However, in a recent Scandinavian study, men showed less initiative to talk about their own impending death compared with women [34]. Interestingly, after open-ended evoking intervention “*What changes can you see taking place in the future*” engagement of males in these talks could be clearly increased [34].

The observed gender effects might result from gender-associated differences in communication style, socialization, information needs, understanding of own disease and prognosis, and their approach to decision-making. Men generally seek professional help and/or psychological support less frequently than women do. Men are less likely to recognize and label feelings and emotional problems [35–37] and show more difficulties in dealing with intimacy [37]. The product of masculine gender-role socialization results in the ideological position that men should be tough, competitive, and emotionally inexpressive [35, 36]. These psychological and social factors may hinder the access to difficult and emotional loaded topics such as fertility [38], death, and dying. In fact, in the present study, males reported fewer conversations about death and dying if they were anxious about their own EOL.

Additionally, female gender is known to be linked to higher needs for information, a more active role in decision-making, and a better understanding of disease. Although men are stated to be less informed about psychological aspects, they express fewer needs for information regarding psychological support compared with females [39]. In the present study, females reported significantly more EOL discussions with a psychologist and other cancer patients. Other studies show that female cancer patients report more discussions about life expectancy with their oncologist, are more likely to understand the incurability of their disease, play a more active role in decision-making, and desire more emotional support from their oncologists compared with male cancer patients [40, 41]. Otherwise, the lower need for EOL conversations of males might result from a less precise conception of desired EOL care or lower need to limit medical interventions in their last period of life. In fact, females were more likely to refuse treatment at EOL [42] and to have early DNR orders [43], refusing to die in a hospital [44]. In the daily clinical routine, there seems to be a high need for an easy access to EOL conversations, especially for male cancer patients to enable a less aggressive and more consistent end-of-life care. Therefore, further research is needed to develop and investigate gender-sensitive approaches to EOL conversations.

## Conclusions

The influence of gender and the strong tendency to initiate EOL conversations as late as possible hinder the access to EOL conversations in time and might disadvantage males. Therefore, we recommend a two-step approach to EOL discussions to achieve a more gender-sensitive approach. This two-step approach might lead to a better balance between the risk of premature stressful discussions on the one hand and missing the right time point on the other: (1) an *obligatory* “early” talk offering basic information about the necessity of EOL conversations or ACP in general, including information about the probability of (unexpected) disease-associated incapability; (2) Later invitations to discuss specific EOL issues or proceed ACP with a gender-sensitive approach either using open-ended, non-confronting, and non-provocative questions (e.g., “*What changes can you see taking place in the future*”) or using starting points in medical care or organizational “facts” particularly for men.

## Limitations

Next to the known general limitations of cross-sectional surveys, such as deductive assumptions in the conception of the questions, the study has several additional limitations. The study group is not representative for cancer patients in general and the data could not be simply transferred to other non-clinical oncology settings. The refusal rate in our study is relatively high and the main reasons not to participate were fear of emotional burden and avoidance to talk about end-of-life issues, reflecting a general reluctance to talk about EOL issues. Therefore, the study group likely represents patients who are generally rather disposed to talk about end-of-life issues. Nevertheless, our refusal rate is comparable with other studies in this field [45]. However, it could not be excluded that due to a high selection bias all participating females have psychologically a more unimpeded access to conversations about end-of-life issues. In addition, of course, the approach to end-of-life conversations notably needs always to be individual and context-sensitive.
